# A longitudinal study of polygenic score and cognitive function decline considering baseline cognitive function, lifestyle behaviors, and diabetes among middle-aged and older US adults

**DOI:** 10.1186/s13195-023-01343-1

**Published:** 2023-11-10

**Authors:** Tingting Liu, Changwei Li, Ruiyuan Zhang, Eugenia Flores Millender, Hongyu Miao, Michael Ormsbee, Jinzhen Guo, Adrianna Westbrook, Yang Pan, Jing Wang, Tanika N. Kelly

**Affiliations:** 1 College of Nursing, Florida State University, Tallahassee, FL 32306 USA; 2 Department of Epidemiology, Tulane University School of Public Health and Tropical Medicine, 1440 Canal Street Suite 2000, New Orleans, LA 70112 USA; 3 Center of Population Sciences for Health Equity, Florida State University College of Nursing, Tallahassee, FL 32306 USA; 4 Institute of Sports Sciences and Medicine, Florida State University, Tallahassee, FL 32306 USA; 5 Department of Radiation Oncology, University of Texas Southwestern Medical Center, Dallas, TX 75390 USA; 6 Department of Pediatrics, Emory University School of Medicine, Atlanta, GA 30322 USA; 7 Division of Nephrology, Department of Medicine, University of Illinois at Chicago, Chicago, IL 60612 USA

**Keywords:** Polygenic score, Cognitive function decline, Lifestyle, Interaction

## Abstract

**Background:**

Genomic study of cognition decline while considering baseline cognition and lifestyle behaviors is scarce. We aimed to evaluate the impact of a polygenic score for general cognition on cognition decline rate, while considering baseline cognition and lifestyle behaviors, among the general population and people with diabetes, a patient group commonly affected by cognition impairment.

**Methods:**

We tested associations of the polygenic score for general cognition with annual changing rates of cognition measures in 8 years of follow-up among 12,090 White and 3100 Black participants of the Health and Retirement Study (HRS), a nationally representative sample of adults aged 50 years and older in the USA. Cognition measures including word recall, mental status, and total cognitive score were measured biannually. To maximize sample size and length of follow-up, we treated the 2010 wave of survey as baseline, and follow-up data until 2018 were analyzed. Baseline lifestyle behaviors, *APOE* status, and measured cognition were sequentially adjusted. Given racial differences in polygenic score, all analyses were conducted by race.

**Results:**

The polygenic score was significantly associated with annual changing rates of all cognition measures independent of lifestyle behaviors and APOE status. Together with age and sex, the polygenic score explained 29.9%, 15.9%, and 26.5% variances of annual changing rates of word recall, mental status, and total cognitive scores among Whites and explained 17.2%, 13.9%, and 18.7% variance of the three traits among Blacks. Among both White and Black participants, those in the top quartile of polygenic score had the three cognition measures increased annually, while those in the bottom quartile had the three cognition measures decreased annually. After further adjusting for the average cognition assessed in 3 visits around baseline, the polygenic score was still positively associated with annual changing rates of all cognition measures for White (*P* ≤ 2.89E − 19) but not for Black (*P* ≥ 0.07) participants. In addition, among participants with diabetes, physical activity offset the genetic susceptibility to decline of mental status (interaction *P* ≤ 0.01) and total cognitive scores (interaction *P* = 0.03).

**Conclusions:**

Polygenic score predicted cognition changes in addition to measured cognition. Physical activity offset genetic risk for cognition decline among diabetes patients.

**Supplementary Information:**

The online version contains supplementary material available at 10.1186/s13195-023-01343-1.

## Introduction

According to the most recent national survey, mild cognitive impairment and dementia affected as many as 22% and 10% of individuals aged 65 years and older in the USA [1]. The two types of cognitive dysfunction are even more prevalent among persons with diabetes [2, 3]. As aging of the US population, the burden of cognitive dysfunction is projected to increase dramatically [4]. Persons with cognitive dysfunction gradually lose ability to independently perform daily activities. This not only impairs their quality of life but also places a huge burden on caregivers [5]. Most types of cognitive dysfunction cannot be cured. Thus, there is an urgent need to identify those who are at high risk for cognitive dysfunction before onset or at early stage so that primordial prevention can be implemented to maintain and improve cognitive function.

Genetic factors play an important role in cognitive function. The heritability estimates are as high as 20–50% for general cognition [6, 7] and 58–79% for late onset Alzheimer’s disease (AD) [8]. Although *APOE* ɛ4 and ɛ2 alleles are driving forces of AD and its related dementia, the burden of other risk alleles with smaller effects is also important for AD and dementia [9, 10]. It has been shown that *APOE* predicts AD risk better at younger ages, while other risk alleles predict AD risk better in older ages [11, 12]. In some ancestral groups, *APOE* ɛ4 and ɛ2 had weak or even no associations with cognitive dysfunction [13, 14]. The current polygenic scores (PGSs) combining millions of variants across the genome can identify AD cases with high accuracy, with area under the curves (AUC) reaching 0.74 or higher [11, 15]. PGSs can also help identify individuals who are most likely to have cognitive function decline [16, 17]. In the Alzheimer’s Disease Neuroimaging Initiative, PGS detected 72.8% of individuals whose cognitive function declined by 15 points in 4 years of follow-up [16]. In a 2017 study, Marden and colleagues discovered that a PGS comprised of 22 AD-associated loci predicted faster memory decline in 14 years [18]. However, it is unclear whether PGSs predict cognitive function decline in addition to measured cognitive function at baseline.

About 40% of cognitive dysfunction cases are attributable to modifiable risk factors [19], and primordial prevention strategies, such as lifestyle modification, targeting at the modifiable risk factors are recommended by the current practice guideline [20]. In the USA, the largest proportion of AD cases is attributable to a lack of physical activity (PA) [21]. Despite the bulk of evidence suggesting that PA improves cognitive function [22], there is significant variability in individual response to PA on cognitive outcomes. Such variability may be driven by genetic factors [23–32]. Investigating the joint effect of lifestyle behaviors and genetic factors may help develop targeted intervention strategies for cognitive dysfunction.

The current study examined associations of a PGS for general cognition with changes of cognitive function over 8 years of follow-up while considering baseline cognitive function, lifestyle behaviors, and diabetes. The primary goal was to evaluate whether PGS predicts changes of cognitive function in addition to baseline measures of cognitive function, with a secondary goal of identifying population subgroups who may benefit more from lifestyle modification based on their genomic profiles.

## Methods

### Study design

This was a prospective cohort study based on panel data from the Health and Retirement Study (HRS). The HRS has surveyed a representative sample of more than 26,000 Americans over the age of 50 every 2 years since 1992 [33]. To maximize sample size and follow-up time, we treated the 10th wave of survey conducted in 2010 as baseline, and a total of 15,190 HRS participants with available PGS for general cognition were included in the cross-sectional analyses. Among these participants, 6300 had at least one follow-up visit in 8 years till 2018 and were included in the longitudinal analyses.

### Genotyping, PGS calculation, and *APOE* isoforms

Genome-wide genotypes were assayed using the Illumina’s Human Omni2.5-Quad (Omni2.5) BeadChip methodology [34]. After stringent quality control, genotype data was imputed to the 1000 Genome Project cosmopolitan reference panel phase 3 version 5, and ancestry-specific genetic principal components (PCs) were calculated. PGS for general cognition was developed based on results from a genome-wide association studies (GWAS) meta-analysis among 300,486 individuals of European ancestry [35]. The PGS was calculated by combining cognitive function increasing alleles of single nucleotide polymorphisms (SNPs) weighted by reported effect sizes using the PRSice and PLINK software [36, 37]. SNPs was not trimmed by linkage disequilibrium or filtered by *P*-value thresholding [37]. To avoid overfitting, SNP weights were estimated after removing HRS from the GWAS meta-analysis. Additionally, five cohorts requested their results to be removed due to data use restriction. After removing the six cohorts, the updated GWAS meta-analysis has a sample size of 274,774. The PGS contained 1,382,609 variants and was standardized within ancestry to have a mean = 0 and standard deviation (SD) = 1. Details of genotype quality control and PGS calculation are reported in Supplementary Methods.

*APOE* isoforms were determined by predesigned TaqMan allelic discrimination SNP arrays for 17,237 HRS participants and were inferred for 1956 participants based on the imputed dosage data of rs7412 and rs429358.

### Cognitive function measurement and calculation of annual changing rates

Cognitive function measures included mental status and word recall and were tested every 2 years for five waves of data collection from 2010 to 2018. The test battery included administration of serial 7 s, counting backwards, object naming test, recall of the date and the US president and the vice-president, and word recall to reflect participants’ episodic memory, knowledge, attention, language, and orientation [38]. Details of these tests are provided in Supplementary Methods. A total cognitive function score summarizing mental status and word recall was also provided by the HRS. The total cognitive function score ranged from 0 to 35, with a higher score indicating better cognitive function.

*Annual changing rates of cognitive function measures* during 8 years of follow-up were estimated for 5382 European American (EA) and 920 African American (AA) participants with at least one follow-up visit. The changing rates were calculated by a mixed effect model with follow-up time treated as random effects. The estimated annual changing rates were normalized to a mean = 0 and SD = 1.

### Diabetes, education, and lifestyle behaviors

Diabetes status, years of education, and lifestyle behaviors including cigarette smoking, alcohol drinking, and PA were self-reported in the 10th wave of survey. Information on leisure-time and work-related light-, moderate-, and vigorous-intensity PA for the past 12 months was collected. Consistent with prior studies [39, 40], the frequency of PA by each intensity was coded as follows: 0 = hardly ever or never, 1 = 1–3 times a month, 2 = once a week, 3 = more than once a week, and 4 = every day. An index score was created as the sum of frequencies of all PA components.

### Statistical analyses by ancestral groups

Cross-sectional associations between the PGS and cognitive function measures in wave 10 were tested using four linear regression models: a genomic only model adjusting for ancestry information (the first 3 genetic PCs for EAs and the first 10 genetic PCs for AAs) and three additional models sequentially adding (1) age and sex, (2) *APOE* isoforms, and (3) education and lifestyle behaviors. Variance (*R*^2^) explained by all variables was reported for each linear regression model. Bonferroni correction for 12 tests (3 cognition scores × 4 models) was used to determine significant associations in each ancestral group.

Associations between the PGS and annual changing rates of cognitive function measures were examined using five linear regression models: a genomic only model adjusting for ancestry information (the first 3 PCs for EAs and the first 10 PCs for AAs) and four additional models sequentially adding (1) age and sex, (2) baseline cognitive function measures, (3) *APOE* isoforms, and (4) education and lifestyle behaviors. We also built a model adjusting for age, sex, genetic ancestry, and mean cognitive function measures in waves 8, 9, and 10. Variance (*R*^2^) explained by all variables was reported for each linear regression model. Bonferroni correction for 18 tests (3 cognition scores × 6 models) was used to determine significant associations in each ancestral group.

To explore age group differences, we divided HRS participants by the median age and tested associations of PGS with annual changing rates of cognitive function measures by age groups. We also tested interactions between the PGS and lifestyle behaviors on annual changing rates of cognitive function measures overall and among participants with self-reported diabetes at baseline. Considering that interaction analysis generally has lower power than direct association tests, we used a *p* < 0.05 to define significant interactions. For a significant interaction, we used a sliding window approach to divide participants into high and low genetic risk groups based on a percentile of PGS and visually compared the standardized effect sizes of a lifestyle behavior on annual changing rates of cognitive function measures between the two groups. This allowed us to identify a PGS group that benefited more from lifestyle behavior.

All analyses were conducted using R (version 4.2). A two-sided *P* value < 0.05 was used to define significant associations or interactions.

## Results

A total of 12,090 EAs and 3100 AAs were included in the cross-sectional analyses (Table 1). The average age was 68.56 years among EAs and 62.99 years among AAs. More than half of the participants were females (57.0% in EAs and 61.6% in AAs). Participants had an average of more than 12 years of education. Compared to EAs, AAs had larger BMI (30.28 vs. 27.97 kg/m^2^), less frequency of PA (11.20 vs. 12.06), and lower scores of word recall (8.84 vs. 10.04), mental status (11.17 vs. 12.90), and total cognitive function (19.80 vs. 22.51). However, the annual changing rate of total cognitive function score was similar between the two ancestral groups (− 0.56 vs. − 0.57). Compared to the baseline sample, participants included in the longitudinal analyses were older and more likely to be females and had fewer years of education (Supplementary Table S1).

**Table 1:** Baseline characteristics and changing rates of the study participants

Variables	European Americans (*N* = 12090)	African Americans (*N* = 3100)
Mean age (SD), years	68.56 (11.61)	62.99 (10.86)
Female (%)	6894 (57.0)	1910 (61.6)
Mean years of education (SD)	13.26 (2.53)	12.18 (2.89)
Ever drinker, *N* (%)	6388 (58.6)	1399 (49.1)
Ever smoker, *N* (%)	6138 (56.6)	1705 (60.1)
Mean body mass index (SD), kg/m^2^	27.97 (5.87)	30.28 (7.00)
Mean physical activity index (SD)	12.06 (4.30)	11.20 (4.16)
Mean baseline cognitive function (SD)		
Word recall	10.04 (3.38)	8.84 (3.25)
Mental status	12.90 (2.17)	11.17 (2.75)
Total cognitive function	22.51 (4.70)	19.80 (5.16)
Mean annual changing rates (SD)		
Word recall	− 0.24 (0.20)	− 0.16 (0.18)
Mental status	− 0.18 (0.14)	− 0.21 (0.21)
Total cognitive function	− 0.57 (0.32)	− 0.56 (0.38)
*APOE* genotypes, *N* (%)		
22	77 (0.6)	35 (1.1)
23	1551 (12.8)	433 (14.0)
24	253 (2.1)	145 (4.7)
33	7306 (60.4)	1428 (46.1)
34	2658 (22.0)	926 (29.9)
44	244 (2.0)	133 (4.3)

*SD* standard deviation

### PGS and cognitive function at baseline

As expected, the PGS for general cognition was positively associated with all cognitive function measures at baseline (wave 10) (Table 2). In the genomic only model, a one SD increase in PGS was associated with 0.545 (*P* < 0.0001), 0.513 (*P* < 0.0001), and 1.031 (*P* < 0.0001) higher scores of word recall, mental status, and total cognitive function among EAs, explaining 2.6%, 5.5%, and 4.7% of variance of the three traits. Such associations had smaller magnitudes, explained less variances, but remained significant among AAs, with a one SD increase in PGS associated with 0.224 (*P* = 0.0004), 0.317 (*P* < 0.0001), and 0.523 (*P* < 0.0001) higher scores of the three traits. Adding age and sex to the genomic model slightly strengthened the associations and increased the model fit *R*^2^ to 21.6%, 11.0%, and 20.7% among EAs and to 12.0%, 10.5%, and 14.6% among AAs (Table 2 and Supplementary Table S2). Further adding *APOE* isoforms to the model had very minor influence on both the effect of PGS and model fit. Education and lifestyle behaviors substantially attenuated the effect of PGS on all cognitive function measures. For example, after further adjusting for education and lifestyle behaviors, the effect size of PGS on total cognitive function score dropped by 28.0% from 1.093 (*P* < 0.0001) to 0.787 (*P* < 0.0001) among EAs and by 36.2% from 0.531 (*P* < 0.0001) to 0.339 (*P* = 0.0003) among AAs.

**Table 2:** Associations of polygenic score for general cognition with baseline cognitive function measurements

**Models**	**European Americans**			**African Americans**		
	**Beta (SE)**	***P***	***R***^**2**^	**Beta (SE)**	***P***	***R***^**2**^
*Word recall*						
Base model	0.545 (0.033)	< 0.0001	0.026	0.224 (0.063)	0.0004	0.009
Base model + age and sex	0.591 (0.030)	< 0.0001	0.216	0.220 (0.060)	0.0002	0.120
Base model + age, sex, and APOE	0.588 (0.030)	< 0.0001	0.219	0.221 (0.060)	0.0002	0.121
Full model	0.412 (0.029)	< 0.0001	0.279	0.137 (0.057)	0.02	0.207
*Mental status*						
Base model	0.513 (0.024)	< 0.0001	0.055	0.317 (0.059)	< 0.0001	0.022
Base model + age and sex	0.526 (0.023)	< 0.0001	0.110	0.320 (0.057)	< 0.0001	0.105
Base model + age, sex, and APOE	0.525 (0.023)	< 0.0001	0.112	0.319 (0.057)	< 0.0001	0.108
Full model	0.385 (0.023)	< 0.0001	0.195	0.211 (0.051)	< 0.0001	0.278
*Total cognitive function*						
Base model	1.031 (0.053)	< 0.0001	0.047	0.523 (0.112)	< 0.0001	0.018
Base model + age and sex	1.097 (0.048)	< 0.0001	0.207	0.532 (0.104)	< 0.0001	0.146
Base model + age, sex, and APOE	1.093 (0.048)	< 0.0001	0.211	0.531 (0.104)	< 0.0001	0.149
Full model	0.787 (0.046)	< 0.0001	0.305	0.339 (0.094)	0.0003	0.309

Base model adjusted for the first 3 genetic principal components (PCs) for European Americans and the first 10 PCs for African Americans. Full model further adjusted for smoking, drinking, education, and physical activity

*SE* standard error

### PGS and changing rates of cognitive function

**Fig. 1:**
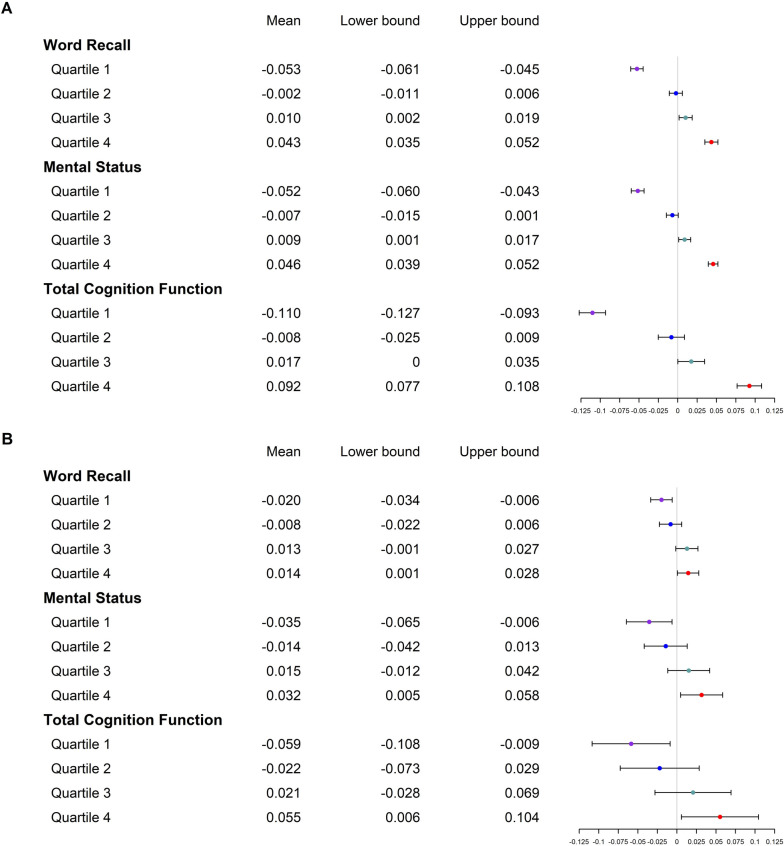
Age, sex, and genetic ancestry adjusted mean annual changing rates of cognitive function measures during 2010 and 2018 according to quartiles of polygenic score for general cognition among European American (**A**) and African American (**B**) participants of the Health and Retirement Study

The PGS for general cognition was positively associated with annual changing rates of all cognitive function measures among EAs (Table 3). Together with age and sex, the PGS explained 29.9%, 15.9%, and 26.5% of the variances of annual changing rates of word recall, mental status, and total cognitive function. Participants in the top quartile of PGS had word recall, mental status, and total cognitive function scores increased by 0.043, 0.046, and 0.092 annually, while those in the bottom quartile of PGS had the three cognitive function measures decreased by 0.053, 0.052, and 0.110 annually (Fig. 1A). After further adjusting for baseline cognitive function, the PGS was still significantly associated with annual changing rates of all cognitive function measures, although the magnitudes of associations were dramatically attenuated. Even after adjusting for the average cognitive function measured in waves 8, 9 and 10, such associations remained significant. *APOE* isoforms, education, and lifestyle behaviors had minimum influence on the associations of PGS with changing rates of cognitive function measures.

**Table 3:** Associations of polygenic score for general cognition with annual changing rates of the cognitive function measurements over 8 years of follow-up

Models	European Americans			African Americans		
	**Beta (SE)**	***P***	***R***^**2**^	**Beta (SE)**	***P***	***R***^**2**^
***Word recall***						
Genomic model	0.038 (0.002)	< 0.0001	0.035	0.013 (0.004)	0.0004	0.010
Genomic, age and sex adjusted base model	0.041 (0.002)	< 0.0001	0.299	0.012 (0.003)	0.0004	0.172
Base model + baseline word recall	0.016 (0.001)	< 0.0001	0.673	0.004 (0.002)	0.07	0.588
Base model + mean word recall	0.012 (0.001)	< 0.0001	0.708	0.003 (0.002)	0.19	0.616
Base model + baseline word recall, and APOE	0.016 (0.001)	< 0.0001	0.674	0.004 (0.002)	0.07	0.589
Full model	0.012 (0.001)	< 0.0001	0.692	0.002 (0.002)	0.35	0.619
***Mental status***						
Genomic model	0.039 (0.002)	< 0.0001	0.072	0.024 (0.007)	0.0008	0.051
Genomic, age and sex adjusted base model	0.040 (0.002)	< 0.0001	0.159	0.024 (0.007)	0.0005	0.139
Base model + baseline mental status	0.012 (0.001)	< 0.0001	0.662	0.003 (0.004)	0.43	0.765
Base model + mean mental status	0.010 (0.001)	< 0.0001	0.659	0.004 (0.004)	0.35	0.758
Base model + baseline mental status, and APOE	0.012 (0.001)	< 0.0001	0.666	0.003 (0.004)	0.41	0.767
Full model	0.010 (0.001)	< 0.0001	0.675	0.001 (0.004)	0.72	0.784
***Total cognitive function***						
Genomic model	0.082 (0.004)	< 0.0001	0.064	0.044 (0.013)	0.0006	0.039
Genomic, age and sex adjusted base model	0.085 (0.004)	< 0.0001	0.265	0.043 (0.012)	0.0003	0.187
Base Model + baseline total cognitive function	0.024 (0.003)	< 0.0001	0.704	0.008 (0.007)	0.25	0.751
Base Model + mean total cognitive function	0.017 (0.003)	< 0.0001	0.715	0.005 (0.007)	0.45	0.762
Base Model + baseline total cognitive function, and APOE	0.024 (0.003)	< 0.0001	0.708	0.008 (0.007)	0.26	0.754
Full model	0.019 (0.003)	< 0.0001	0.722	0.005 (0.007)	0.48	0.776

Full model adjusted for age, sex, ancestry, baseline cognitive function, *APOE*, smoking, drinking, education, and physical activity

*SE* standard error

The PGS was also positively associated with annual changing rates of cognitive function measures among AAs (Table 3 and Fig. 1B). Age, sex, and genomic information explained 17.2%, 13.9%, and 18.7% of variance of annual changing rates of word recall, mental status, and total cognitive function (Table 3 and Supplementary Table S2). However, after adjusting for baseline cognitive function, the PGS was no longer associated with changing rates of any cognitive function measures (*P* ≥ 0.07).

### PGS and changing rates of cognitive function by age groups

Age modified the effect of PGS on annual changing rates of word recall among EAs (interaction *P* = 0.01). As shown in Supplementary Figure S1, PGS for general cognition was more strongly associated with word recall among EAs aged less than 73 years (beta = 0.044, *P* < 0.0001) than those older than 73 years (beta = 0.035, *P* < 0.0001).

### Interplay of lifestyle behaviors and PGS on changing rates of cognitive function

**Fig. 2:**
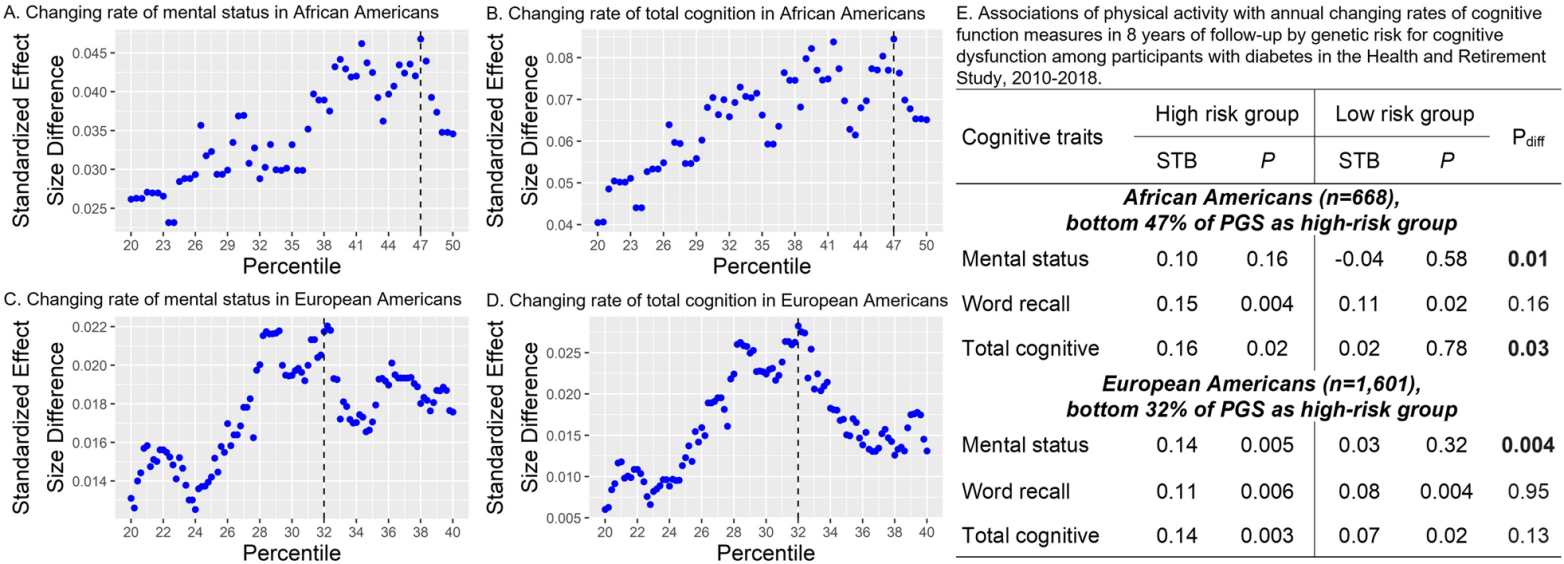
Differences of age, sex, and ancestry adjusted standardized effect sizes of physical activity between diabetes participants below and above a percentile of polygenic scores on annual changing rates of mental status and total cognitive function during 2010 and 2018 among African American (**A** and **B**) and European American (**C** and **D**) participants in the Health and Retirement Study, and age, sex, education, body mass index, smoking, drinking, and ancestry adjusted standardized effect sizes of physical activity (**E**)

We identified significant interactions of PA with PGS on changing rates of cognitive function measures among 668 AA and 1601 EA participants with diabetes. Specifically, PA modified the effects of PGS on annual changing rates of mental status (interaction *P* = 0.01) and total cognitive function (interaction *P* = 0.03) among AA participants with diabetes and on mental status (interaction *P* = 0.004) among EA participants with diabetes. As shown in Fig. 2A–D, the difference of standardized beta coefficients (STB) of PA on annual changing rates of the two cognitive measures peaked at 47% of PGS for AA participants and at 32% of PGS for EA participants. Therefore, we defined high-risk groups as those in the bottom 47% and 32% of PGS for AA and EA participants, respectively, based on findings on mental status and total cognitive score. After adjusting for age, sex, education, BMI, cigarette smoking, alcohol drinking, and ancestry information, STB of PA on annual changing rate of total cognitive function was 0.16 (*P* = 0.02) in the high-risk group and 0.02 (*P* = 0.78) in the low-risk group among AA participants (Fig. 2E). Similarly, among EA participants, STB of PA on annual changing rate of mental status was 0.14 (*P* = 0.005) in the high-risk group and 0.03 (*P* = 0.32) in the low-risk group (Fig. 2E). No lifestyle behavior modified the effect of PGS among the overall participants.

## Discussion

In this nationally representative sample of middle-aged and older US adults, we identified that PGS for general cognition was associated with both baseline and longitudinal changes of cognitive function measures in 8 years of follow-up. The associations were independent of *APOE* isoforms, education, and lifestyle behaviors. Furthermore, even after adjusting for the average cognitive function measured during 3 surveys in 4 years around baseline, the PGS still significantly predicted changes of cognitive function measures among EAs but not among AAs. The PGS explained more variation of both baseline and annual changing rates of cognitive function measures among EAs than AAs. We also discovered that among participants with diabetes, those having a genetically determined low cognitive function benefited more from PA in maintaining cognitive function during follow-up. These findings may help to develop targeted intervention.

PGS for general cognition was significantly associated with the annual changing rates of all cognitive function measures even after adjusting for the average of cognitive function measured during 3 consecutive surveys in 4 years around baseline among EAs. The finding suggests that PGS for general cognition captured additional information, such as long-term burden of cognitive dysfunction, compared to single measures of cognitive function. While this is the first report of added values of PGS in addition to measured cognitive function, similar phenomenon has been observed for other complex traits. For example, in the UK Biobank, even after adjusting for low-density lipoprotein cholesterol (LDLC) level, a PGS for LDLC was still significantly associated with risk of ischemic heart disease [41]. Our findings provide rationale for using PGS along with measured cognitive function to predict cognitive function decline for older US adults. It is noted that education and lifestyle behaviors attenuated the effect sizes of PGS, suggesting that lifestyle modification may have moderated levels of some mediators through which PGS exerted an effect on cognitive function. Finally, a one SD lower PGS was associated with 0.08 SD faster annual declining rate of the total cognition. In a previous study among 1049 elderly Catholic clergy members participating in the Religious Orders Study with a similar length of follow-up (15 years), a subgroup with slow cognition decline had an annual declining rate of − 0.04 SD [42]. Our reported effect size is equivalent to declining about two times as fast as the slow decline group in the ROS study.

Among individuals with diabetes, we demonstrated that PA ameliorated the genetic susceptibility to cognitive function decline. We further identified two cutoff points of PGS to define high-risk groups for AAs and EAs with diabetes respectively. Cognitive dysfunction affects up to 45% of persons with type 2 diabetes mellitus [2]. Despite the bulk of evidence suggesting that aerobic exercise improves cognitive function [22], there is significant variability in individual response to exercise programs on cognitive outcomes. Such variability may be driven by genetic factors [23]. This hypothesis has been tested in several candidate genes studies with inconsistent findings. For example, while individuals with *BDNF* Val/Val genotype have been shown to benefit exclusively from cognition-enhancing effects of exercise in some studies [24–26], others found that Met allele carriers were more likely to offset poor cognitive performance by engaging in higher levels of exercise [27, 28]. Likewise, compared to non-*APOE* ε4 carriers, larger cognitive benefits related to exercise are typically [29–31], but not always [32], reported in *APOE* ε4 carriers. However, to our knowledge, no prior studies incorporated PGS, which is a much more comprehensive indicator of genetic risk [43]. Our findings may help to develop targeted intervention strategies to prevent cognitive dysfunction for diabetes patients.

The performance of PGS in AA participants was less optimal than EA participants. It is a well-recognized problem among PGSs derived from GWAS conducted in primarily European samples [44–46]. Future large-scale GWAS on cognitive function among participants of African ancestry are needed to develop better PGS for this ancestry group. Our study provided the first evidence that PGS for general cognition was more strongly associated with the changing rates of words recall among EAs aged less than 73 years than those aged 73 years and older. This may be because very old adults already had low cognitive function, and there was little room for changes.

The current study has several notable strengths. First, the HRS is a nationally representative survey of middle-aged and older US adults. Our findings have high generalizability. Second, cognitive function was repeatedly assessed during follow-up. This allowed us to pinpoint the impact of PGS on changing rates of cognitive function. Third, the inclusion of both AAs and EAs provided an opportunity to investigate racial differences in the performance of PGS for cognitive function. Our study also has limitations. The PGS was developed based on GWAS conducted predominantly among participants of European ancestry. Its performance for AA participants was less optimal. Second, sample size for AAs was relatively small. We may have less power to detect a significant effect of PGS on changing rates of cognitive function after adjusting for baseline cognitive function. Newer waves of the HRS data will be available, and an updated analysis with the new data in the future will increase statistical power and may yield more significant findings, particularly among AA participants. Third, PGS for Hispanic participants of the HRS is not available. Therefore, we were not able to evaluate its impact on cognition changing rates in this ethnic group. Fourth, although adding genomic information to age and sex adjusted models improved model fit substantially, the overall model fit *R*^2^ was still below 0.3. Additional genomic loci for general cognition and better polygenic score algorithms are needed to better characterize genomic risk of cognitive impairment. Finally, physical activity was based on self-report and may be subject to information bias. However, previous studies have shown that as long as misclassifications of the two variables in an interaction term are not correlated, the bias has minimum influence on interaction test [47]. In the current study, genotyping is very objective and has very high accuracy, and its measurement error is less unlikely to be correlated with that of physical activity. Therefore, our interaction test should be not or minimally influenced by the self-reported nature of physical activity.

To conclude, we identified that a PGS for general cognition predicted longitudinal changes of cognitive function in addition to measured cognitive function at baseline, and among diabetes patients, PA ameliorated the genetic susceptibility to cognitive function decline.

### Supplementary Information

**Additional file 1: Supplementary Methods.** Genotyping, quality control, and PRS calculation. Cognitive function tests administered in the HRS. **Supplementary Table S1.** Characteristics of participants included in the analyses and removed from the analyses of changing rate. **Supplementary Table S2.** Model fits before and after adding genomic information to age and sex adjusted models for baseline and annual changing rates of cognition measures by race. **Supplementary Figure S1.** Subgroup Analyses of the Associations of Polygenic Risk Score with Changing Rate of Cognitive Function Measurements over 6 years Follow-up among EA by Age Groups.

## Data Availability

The HRS data is publicly available at https://hrs.isr.umich.edu/data-products.
