# Supplementary material for: A longitudinal study of polygenic score and cognitive function decline considering baseline cognitive function, lifestyle behaviors, and diabetes among middle-aged and older US adults
Source: Alzheimers Res Ther. 2023 Nov 10;15:196. doi: 10.1186/s13195-023-01343-1 (PMC10636974; doi:10.1186/s13195-023-01343-1)
Supplement: Supplementary file 1 — Additional file 1: Supplementary Methods. Genotyping, quality control, and PRS calculation. Cognitive function tests administered in the HRS. Supplementary Table S1. Characteristics of participants included in the analyses and removed from the analyses of changing rate. Supplementary Table S2. Model fits before and after adding genomic information to age and sex adjusted models for baseline and annual changing rates of cognition measures by race. Supplementary Figure S1. Subgroup Analyses of the Associations of Polygenic Risk Score with Changing Rate of Cognitive Function Measurements over 6 years Follow-up among EA by Age Groups. [file 13195_2023_1343_MOESM1_ESM.docx]

**The impact of a polygenic score on cognitive function decline considering baseline cognitive function, lifestyle behaviors and diabetes among middle-aged and older US adults**

Tingting Liu, RN, PhD^1*^; Changwei Li, MD, PhD^2*^; Ruiyuan Zhang, MD, MS^2*^; Eugenia Flores Millender, PhD^1,3^; Hongyu Miao, PHD^1^; Michael Ormsbee, PhD^4^; Jinzhen Guo, PhD^5^; Adrianna Westbrook, MS^6^; Yang Pan, PhD^7^; Jing Wang, RN, PhD^1^; Tanika N. Kelly, PhD^7^

1 College of Nursing, Florida State University, Tallahassee, FL 32306, USA.

2 Department of Epidemiology, Tulane University School of Public Health and Tropical Medicine, New Orleans, LA 70112, USA.

3 Center of Population Sciences for Health Equity, Florida State University College of Nursing, Tallahassee, FL 32306, USA.

4 Institute of Sports Sciences and Medicine, Florida State University, Tallahassee, FL 32306, USA.

5 Department of Radiation Oncology, University of Texas Southwestern Medical Center, Dallas, TX 75390, USA.

6 Department of Pediatrics, Emory University School of Medicine, Atlanta, GA 30322, USA.

7 Division of Nephrology, Department of Medicine, University of Illinois at Chicago, Chicago, IL 60612, USA.

*Contribute equally

**Supplementary materials**

**Supplementary methods**

1. **Genotyping, quality control, and PRS calculation.**

Genome-wide genotypes were assayed from saliva samples in prior waves of surveys between 2006 and 2012. The genotyping was performed by the Center for Inherited Disease Research at the National Institutes of Health, using the Illumina’s Human Omni2.5-Quad (Omni2.5) BeadChip methodology.^1^ As described previously,^2,3^ genotyping quality control included HapMap control samples to examine the rate of concordance with HapMap data, blind duplicates for reproducibility, and cross-study duplicates for cross-study reproducibility. Data were checked for chromosomal anomalies, gender mis-annotation, relatedness between each pair of participants, population structure, batch effects, missing call rates, Hardy-Weinberg equilibrium (HWE), duplicate sample discordance, mendelian errors, sample quality, sample exclusion and filtering, SNP filtering, preliminary association tests, and minor allele frequency.^4^ SNPs with HWE p value < 0.0001 or missing call rate > 2% were removed.^4^ After quality control, genetic ancestry was identified through principal component (PC) analysis on genome-wide autosomal SNPs with a minor allele frequency>5% and not in the 2q21, HLA, 8p23 or 17q21.31 regions. After confirming ancestry, PC analysis was run again within each ancestry group to estimate ancestry-specific PCs.

The PRS for general cognition was developed by the HRS research committee using results from a 2018 GWAS meta-analysis^5^ conducted by the Cohorts for Heart and Aging Research in Genomic Epidemiology, the Cognitive Genomics Consortium, and UK Biobank. This meta-analysis included 300,486 individuals aged 16-102 years old of European ancestry and identified 434 independent variants at 148 loci for general cognition. Due to heterogeneity in the measurement of cognition across cohorts in the original GWAS meta-analysis, the first unrotated principal component (FUPC) of all cognition data in each study was used as the general cognition. In the HRS, the FUPC accounted for 37.8% of the total test variance, and loadings on the FUPC were as follows: animal fluency=0.664, number series=0.735, delayed recall=0.624, serial 7’s =0.632, and backward counting=0.346.^6^ The PRS was calculated by combining cognitive function increasing alleles of single nucleotide polymorphisms (SNPs) overlapped with the GWAS meta-analysis weighted by reported effect sizes using PRSice and PLINK software.^7,8^ Based on empirical evidence from studying a series of polygenic scores considering SNP imputation, linkage disequilibrium, SNP p-value thresholding, and methods for SNP correlation estimates, the HRS investigation team discovered that including all available SNPs in a polygenic score without accounting for LD or p-value thresholding either showed larger predictive power or showed similar performance as those considering some degree of LD trimming or p-value thresholding. Therefore, SNPs was not trimmed by linkage disequilibrium or filtered by p-value thresholding in the current polygenic score for general cognition.^8^ SNPs in the MHC region on chromosome 6 (26-33 Mb) were excluded. To avoid overfitting, SNP weights were estimated after removing the HRS from the original GWAS meta-analysis. Additionally, due to data use restriction, several NHLBI cohorts, including the Aging Gene-Environment Susceptibility -Reykjavik Study (AGES), the Atherosclerosis Risk in Communities Study (ARIC), the Cardiovascular Health Study (CHS), the Framingham Heart Study (FHS), and the Genetic Epidemiology Network of Arteriopathy (GENOA) were also removed from the GWAS meta-analysis. The HRS investigation team worked with the CHRGE-COGENT consortia to generated updated summary statistics after removing the six cohorts results from the original GWAS meta-analysis. The final sample size for the updated GWAS meta-analysis was 247,774. The PRS contains 1,382,609 variants and was standardized within ancestry to have a mean=0 and standard deviation (SD)=1.

1. **The following cognitive function tests are administered in the HRS:**

*Serial 7’s.* Participants were asked to do serial subtraction of 7 beginning from the number of 100 for up to 5 times. Each subtraction was scored independently, and scores on this test ranged from 0 to 5. Higher scores indicate better mental status.

*Counting backwards.* Participants were asked to count backwards for 10 continuous numbers beginning with the number 20. They were allowed two trials for this test. Participants received a score of 2 if they were correct on the first trial, 1 if they were incorrect on the first trial but correct on a second trial, and 0 if they were incorrect on both trials. Higher scores indicate better processing speed and attention.

*Object naming test.* Participants were asked to respond to the following two questions: “What do you usually use to cut paper?” “What do you call the kind of prickly plant that grows in the desert?” Participants received a score of 2 if they were correct on both questions, 1 if they were incorrect on either question, or 0 if they were incorrect on both questions. Higher scores indicate better language performance.

*Recall of the date and the U.S. president and the vice-president.* Participants were asked to recall the year, month, day, and day of the week. They were also asked to recall the last name of the current U.S. president and the vice-president. The number of correctly recalled items was graded, with the score ranging from 0 to 6. Higher scores indicate better orientation performance.

*Word recall.* Participants were asked to recall as many nouns as possible immediately after they were read to a total of 10 nouns. They were asked to recall these nouns again after 5 minutes to assess delayed word recall. Correctly recalled words from both tasks were scored, with the score ranging from 0 to 20. Higher scores indicate better memory performance.

**Supplementary Table S1. Characteristics of participants included in the analyses and removed from the analyses of changing rate.**

|  | **EA** |  |  |  | **AA** |  |  |
| --- | --- | --- | --- | --- | --- | --- | --- |
|  | **Kept (N=5382)** | **Removed (N=6708)** | **P** |  | **Kept (N=920)** | **Removed (N=2180)** | **P** |
| Age, Years, Mean (SD) | 74.22 (6.97) | 63.15 (12.55) | <0.001 |  | 72.18 (6.50) | 58.66 (9.75) | <0.001 |
| Female, N (%) | 3181 (59.1) | 3713 (55.4) | <0.001 |  | 579 (62.9) | 1331 (61.1) | 0.346 |
| Education, Years, Mean (SD) | 13.21 (2.51) | 13.31 (2.54) | 0.038 |  | 11.70 (3.04) | 12.39 (2.81) | <0.001 |
| Ever Drinking, N (%) | 2961 (55.5) | 3427 (61.5) | <0.001 |  | 334 (36.6) | 1065 (55.0) | <0.001 |
| Every Smoking, N (%) | 2956 (55.8) | 3182 (57.3) | 0.101 |  | 518 (57.3) | 1187 (61.4) | 0.041 |
| BMI, kg/m^2^, Mean (SD) | 27.80 (5.43) | 28.13 (6.26) | 0.004 |  | 29.65 (6.24) | 30.59 (7.31) | 0.001 |
| Physical Activity, Mean (SD) | 12.09 (4.15) | 12.03 (4.45) | 0.520 |  | 10.89 (4.05) | 11.35 (4.19) | 0.006 |
| PRS, Mean (SD) | 0.03 (1.00) | -0.02 (1.00) | 0.004 |  | 0.02 (1.00) | -0.01 (1.00) | 0.378 |
| Word Recall, Mean (SD) | 9.82 (3.20) | 10.27 (3.54) | <0.001 |  | 8.39 (3.24) | 9.06 (3.23) | <0.001 |
| Mental Status, Mean (SD) | 13.04 (1.96) | 12.67 (2.46) | <0.001 |  | 10.96 (2.72) | 11.30 (2.76) | 0.005 |
| Total Cognition Function, Mean (SD) | 22.73 (4.24) | 22.15 (5.33) | <0.001 |  | 19.28 (5.06) | 20.12 (5.19) | <0.001 |
| APOE, N (%) |  |  | 0.030 |  |  |  | 0.049 |
| 22 | 37 (0.7) | 40 (0.6) |  |  | 13 (1.4) | 22 (1.0) |  |
| 23 | 713 (13.2) | 838 (12.5) |  |  | 128 (13.9) | 305 (14.0) |  |
| 24 | 105 (2.0) | 148 (2.2) |  |  | 41 (4.5) | 104 (4.8) |  |
| 33 | 3286 (61.1) | 4020 (59.9) |  |  | 457 (49.7) | 971 (44.5) |  |
| 34 | 1154 (21.4) | 1504 (22.4) |  |  | 240 (26.1) | 686 (31.5) |  |
| 44 | 87 (1.6) | 157 (2.3) |  |  | 41 (4.5) | 92 (4.2) |  |

Note: AA=African American, EA=European American, PRS=Polygenic Risk Score, SD=Standard Deviation.

**Supplementary Table S2. Model fits before and after adding genomic information to age and sex adjusted models for baseline and annual changing rates of cognition measures by race.**

|  | European Americans | | | African Americans | | |
| --- | --- | --- | --- | --- | --- | --- |
| models | R^2^ | Net  increase | Percentage  increase | R^2^ | Net  increase | Percentage  increase |
| **Models for baseline cognition measures** | | | | | | |
| ***Word recall*** |  |  |  |  |  |  |
| age and sex adjusted | 0.186 | reference | reference | 0.109 | reference | reference |
| age and sex + genomics | 0.216 | 0.030 | 16.1% | 0.120 | 0.011 | 10.1% |
| ***Mental status*** |  |  |  |  |  |  |
| age and sex adjusted | 0.052 | reference | reference | 0.080 | reference | reference |
| age and sex + genomics | 0.110 | 0.058 | 111.5% | 0.105 | 0.025 | 31.3% |
| ***Total cognition*** |  |  |  |  |  |  |
| age and sex adjusted | 0.153 | reference | reference | 0.125 | reference | reference |
| age and sex + genomics | 0.207 | 0.054 | 35.3% | 0.146 | 0.021 | 16.8% |
| **Models for annual changing rates of cognition measures** | | | | | | |
| ***Word recall*** |  |  |  |  |  |  |
| age and sex adjusted | 0.258 | reference | reference | 0.162 | reference | reference |
| age and sex + genomics | 0.299 | 0.041 | 15.9% | 0.172 | 0.010 | 6.2% |
| ***Mental status*** |  |  |  |  |  |  |
| age and sex adjusted | 0.084 | reference | reference | 0.090 | reference | reference |
| age and sex + genomics | 0.159 | 0.075 | 89.3% | 0.139 | 0.049 | 54.4% |
| ***Total cognition*** |  |  |  |  |  |  |
| age and sex adjusted | 0.196 | reference | reference | 0.150 | reference | reference |
| age and sex + genomics | 0.265 | 0.069 | 35.2% | 0.187 | 0.037 | 24.7% |

**Supplementary Figure S1. Subgroup Analyses of the Associations of Polygenic Risk Score with Changing Rate of Cognitive Function Measurements over 6 years Follow-up among EA by Age Groups**


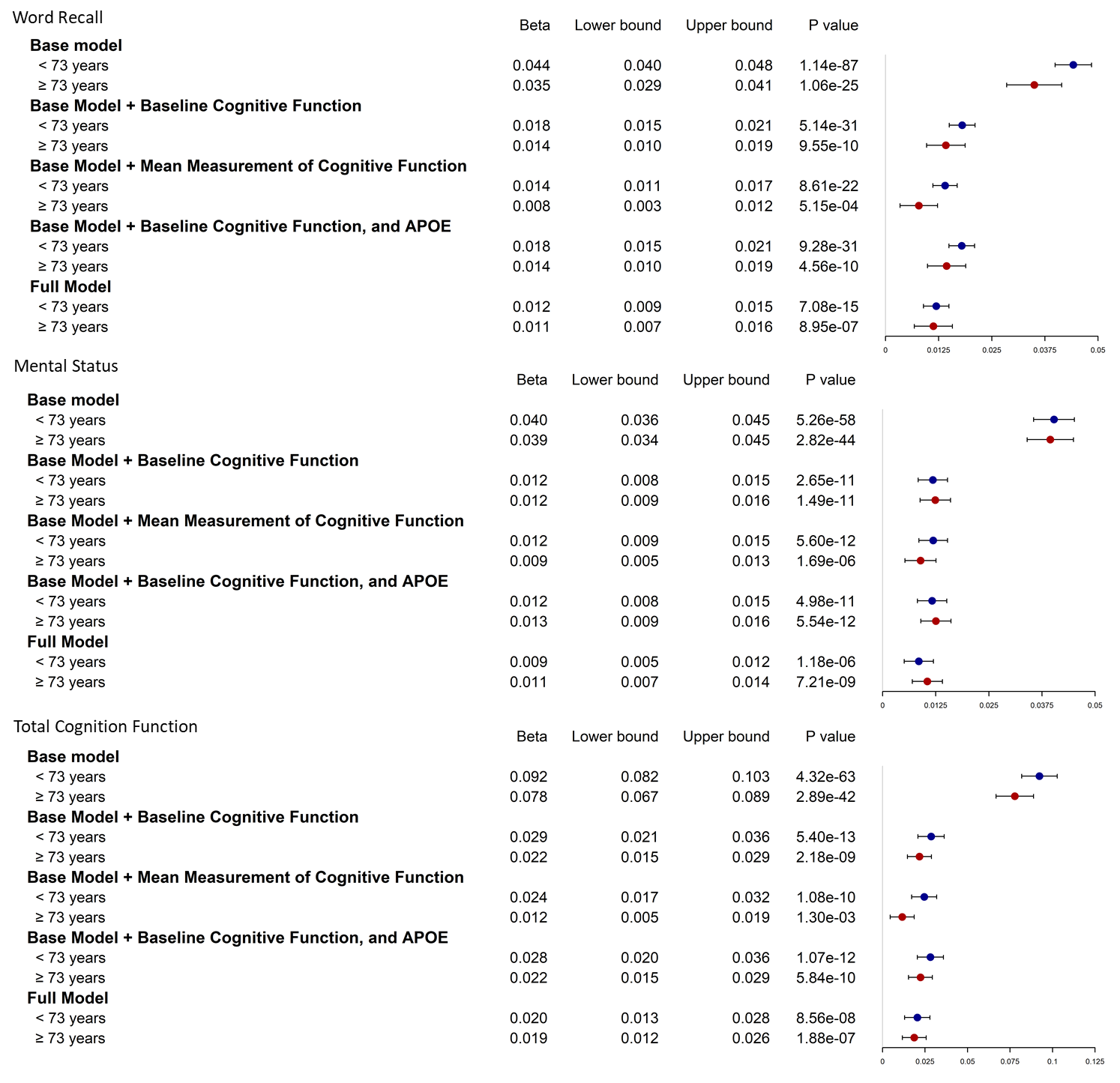


Note: Base model adjusted for age, sex, and PCs, full model further adjusted for smoking, drinking, education, and physical activity in addition to previous model.

EA=European American, PC=Principal Component.

References:

1. Sonnega A, Faul JD, Ofstedal MB, Langa KM, Phillips JW, Weir DR. Cohort profile: The Health and Retirement Study (HRS). *International Journal of Epidemiology*. Apr 2014;43(2):576-585. doi:10.1093/ije/dyu067

2. Liu T, Canon MD, Shen L, et al. The Influence of the BDNF Val66Met Polymorphism on the Association of Regular Physical Activity With Cognition Among Individuals With Diabetes. *Biological Research for Nursing*. Jul 2021;23(3):318-330. doi:10.1177/1099800420966648

3. Liu T, Li H, Conley YP, Primack BA, Wang J, Li C. The Brain-Derived Neurotrophic Factor Functional Polymorphism and Hand Grip Strength Impact the Association between Brain-Derived Neurotrophic Factor Levels and Cognition in Older Adults in the United States. *Biological Research for Nursing*. Apr 2022;24(2):226-234. doi:10.1177/10998004211065151

4. University of Washington. Quality control report for genotypic data. Accessed January 10, 2021, 2021. <https://hrsonline.isr.umich.edu/sitedocs/genetics/HRS2_qc_report_SEPT2013.pdf?_ga=2.85895707.38287890.1610236612-405658027.1610236612&_ga=2.85895707.38287890.1610236612-405658027.1610236612>

5. Davies G, Lam M, Harris SE, et al. Study of 300,486 individuals identifies 148 independent genetic loci influencing general cognitive function. *Nat Commun*. May 29 2018;9(1):2098. doi:10.1038/s41467-018-04362-x

6. Davies G, Lam M, Harris SE, et al. Study of 300,486 individuals identifies 148 independent genetic loci influencing general cognitive function. *Nat Commun*. 05 2018;9(1):2098. doi:10.1038/s41467-018-04362-x

7. Euesden J, Lewis CM, O'Reilly PF. PRSice: Polygenic Risk Score software. *Bioinformatics*. May 01 2015;31(9):1466-8. doi:10.1093/bioinformatics/btu848

8. Ware E, Gard A, Schmitz L, Faul J. *HRS Polygenic Scores-Release 4.3, 2006-2012 Genetic Data*. 2020.
